# Accurate discrimination of bHLH domains in plants, animals, and fungi using biologically meaningful sites

**DOI:** 10.1186/1471-2148-12-154

**Published:** 2012-08-24

**Authors:** Joshua K Sailsbery, Ralph A Dean

**Affiliations:** 1 Fungal Genomics Laboratory, Center for Integrated Fungal Research, Department of Plant Pathology, North Carolina State University, Raleigh, NC, 27606, USA; 2 Bioinformatics Research Center, North Carolina State University, Raleigh, NC, 27606, USA; 3 Centennial Campus, Center for Integrated Fungal Research, North Carolina State University, 851 Main Campus Drive, Suite 233, Raleigh, NC, 27606, USA

**Keywords:** bHLH, Discriminant analysis, Classification, Plants, Animals, Fungi, Environmental sequencing, HMM, Discerning sites, Conserved sites, Variable sites

## Abstract

**Background:**

The highly conserved bHLH (basic Helix-Loop-Helix) domain, found in many transcription factors, has been well characterized separately in Plants, Animals, and Fungi. While conserved, even functionally constrained sites have varied since the Eukarya split. Our research identifies those slightly variable sites that were highly characteristic of Plants, Animals, or Fungi.

**Results:**

Through discriminant analysis, we identified five highly discerning DNA-binding amino acid sites. Additionally, by incorporating Kingdom specific HMMs, we were able to construct a tool to quickly and accurately identify and classify bHLH sequences using these sites.

**Conclusions:**

We conclude that highly discerning sites identified through our analysis were likely under functional constraints specific to each Kingdom. We also demonstrated the utility of our tool by identifying and classifying previously unknown bHLH domains in both characterized genomes and from sequences in a large environmental sample.

## Background

The basic Helix-Loop-Helix (bHLH) domain is a highly conserved peptide sequence across Eukaryotic life that is an essential part of many transcription factors involved in a myriad of regulatory processes, from neurogenesis in mammals [1-4] to environmental response in plants [5]. It provides two of the crucial molecular roles for transcription factors, DNA binding and transcriptional regulation.

The tripartite bHLH domain is ~60 amino acids in length. The DNA-binding region (basic) is located at the N-terminus. This 13 amino acid region is known to bind the hexanucleotide sequence E-box (CANNTG) or its degenerate forms in most bHLH proteins [1]. The two alpha-helices (Helix 1, Helix 2) bind other alpha helices to form homo- or hetero- dimers that stabilize the DNA interaction and promote transcription [6,7]. The two helices contain ~15 amino acids and are separated by a loop of variable length.

Animal bHLH domains form six distinct phylogenetic clades, called groups A-F [8,9]. Group B contains many ancient, highly conserved members such as Myc, Mad, Hairy, and Pho4 [10]. These E-box binders can be identified by the BxR motif at sites 5, 8, and 13, where B is either H or K. Animal group A proteins follow xRx motif at sites 5, 8, and 13 [8]. Members of group A include E12, dHand, MyoD, and Twist which bind the specific E-box sequence CAGCTG or CACCTG. Group E also possess a motif in the basic region, they contain P at site 6, which allows them to bind the E-box degenerate CACGNG sequence. Distinguishing characteristics for groups C and F include the conservation of additional downstream domains, PAS (Per-Arnt-Sim) and COE (Collier/Olf-1/EBF) [9,11], respectively. Finally, group D, which includes members such as Id and Emc, lack any type of basic region and act as transcriptional regulators through hetero-dimerization of basic containing bHLH sequences [12].

From algae to angiosperms, recent work on plants has identified ~33 distinct bHLH domain groups [9,13]. These plant groups are tied to biological functions through characterized members and include processes from light and hormone signaling [14,15] to tissue development [16,17]. Phylogenetic studies have shown that plant bHLH domains are most closely related to animal group B [18,19].

We have recently identified 12 distinct phylogenetic groups in Fungi [20]. Members of these groups have been tied to specific biological roles such as sexual development and glycolysis regulation. Like Plants, fungal bHLH sequences are most closely related to animal group B. Similar to animal groups, the 12 fungal groups can be distinguished with only a few amino acids. For example, only fungal group F4 sequences contain a Y at site 12 in the basic region. Statistical classification analyses led to the identification of the sites that discern between fungal groups.

A primary motivation for this work was to accurately classify and distinguish bHLH sequences from Plant, Animal, and Fungal Kingdoms. There are various types of models that can be utilized for classification. Some, such as decision trees, use solely the amino acid code and ignore physiochemical properties. Moreover, they may have multiple solutions and thus unable to identify sites that are highly important for biological function. Transformation of amino acids into numerical data enables the use of statistical methods such as discriminant analysis. Such analyses provide the means to identify potentially biologically meaningful sites. Atchley et al. [21] developed five numerical indices (factor scores) that allow translation of amino acids from alphabetical to numerical data. These factor scores were derived from Factor Analysis on 495 physiochemical amino acid properties. Each of these independent factor scores (**pah**, **pss**, **ms**, **cc**, **ec**) represent distinct molecular functions of amino acids. Factor score **pah** represents the amino acid properties of polarity, accessibility, and hydrophobicity. The propensity for secondary structure is linked to the **pss** index. Molecular size, codon composition, and electrical charge are represented by the **ms**, **cc**, and **ec** factor scores, respectively.

StepWise Discriminant Analysis (SWDA) and Canonical Variate Analysis (CVA) are two implementations of discriminant analysis [22]. These analyses examine patterns of covariation to obtain a unique classification solution. SWDA and CVA have been used to identify the most discerning sites between animal groups and also between fungal groups [20,23]. These discerning sites are characteristic of the sequences within each group and are often tied to the molecular function of the bHLH domain itself.

Herein, we conducted a study of the discerning sites that exist between plant, animal, and fungal bHLH domains. We built several classification models to discern Kingdom origin and incorporated these into a web-based tool to classify any bHLH sequence. These models provided valuable insight into the fundamental differences between plant, animal, and fungal bHLH domains. We then constructed Kingdom specific HMMs (Hidden Markov Models) and used these to identify bHLH sequences from a large marine environmental sample genome project. Identified sequences were then classified into Kingdoms using our discriminant models. The tool, all source code, models, and relevant data are available at http://www.fungalgenomics.ncsu.edu.

## Results

### Comparing conserved sites in bHLH domains for plants, animals, and fungi

**Figure 1:**
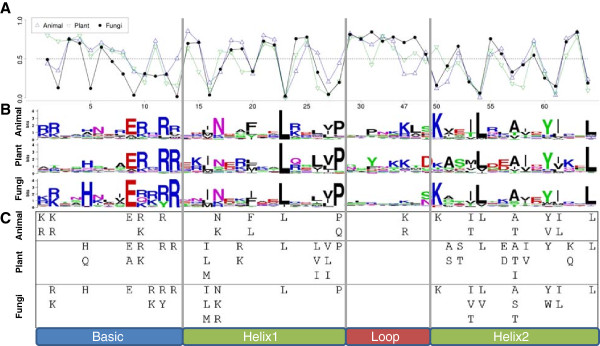
**Animal, Plant, and Fungi bHLH entropies, logos, and consensus.A** The bHLH normalized group entropy by position. Lower values indicate conservation, while values close to one approach unity. **B** The graphical representation of the amino acids at each position of the bHLH domain. Symbols representing amino acids are scaled by their bit score (a derivation of entropy) at a given position. **C** The *50*–*10* consensus sequences for Animal, Plant and Fungi. Using an alignment of bHLH domains within each Kingdom, amino acids occurring at a frequency of more than 50% at a given site are displayed. At each of these sites, additional amino acids are displayed if they are conserved in 10% or more of the sequences. The Plant and Animal consensus sequences are derived from previous work [8,13]. Boundaries between bHLH subdomains are shown at the bottom.

To quantitatively measure the conservation of amino acid sites in the bHLH domain we performed Boltzman-Shannon analyses [8,24,25] for 1302 plant (523), animal (279), and fungal (509) sequences (Figure 1). Within these analyses we grouped like amino acids to account for substitutions of functionally similar amino acids. Normalized group entropy values, ranging from 0–1, indicate conserved to variable sites, respectively. Finally, we visualized the frequency of amino acids per site in bit-score weblogos [26] and provided the published consensus sequences [10,13,20].

Overall, the conservation of amino acids by site of the bHLH was very similar in Plants, Animals and Fungi (Table 1, Figure 1). The pattern of entropy in the three analyses was most similar for the first and second helices. Within the basic region, fungal sequences contained more conserved sites and lower entropy values in general [20].

**Table 1:** Structural attributes and significant sites of the bHLH domain

**Site**	**Structural**	**CS-F**	**CS-A**	**CS-P**	**DT**	**pah**	**pss**	**ms**	**cc**	**ec**	**all**
1	DP										
2	DP	√	*			S			SC	S	S
3											
4											
5	DP	√							SC		S
6	P						S				C
7											
8	DP	*			√						
9	DP	√	*		√						C
10	P	*	*	√					C		C
11	P	*									
12	DP	*	*	√			SC	C			C
13	DP	√		√		SC	SC			S	C
14											
15	P			*						C	C
16	B	√	√	√							
17	P	*	*								
18											
19				*	√						
20	B	*	*	*				C		C	C
21											
22											
23	B	√	√	√		C	C	C			C
24			*								
25											
26		*		√							
27	B	√	√	√						C	C
28	B	√		√							
50	DPB	√	√		√	SC		C	C		SC
51			√	*						C	C
52											
53	B	√	*	√			C				C
54	B	√	√	√		C				C	C
55								S			
56					√					S	S
57		*	*	√							C
58				√							
59											
60		√	*	*							
61	B	√	√	√							C
62											
63											
64	B	√	√	√							

NOTE.–The molecular architechure of bHLH positions is compiled from previous work on crystalline structures of animal proteins. Structural attributes noted are DNA contact of the E-box (D), phosphate backbone contact (P), or buried site within the hydrophobic core of the dimerized helicies (B). Highly (√) and moderately (*) conserved sites are denoted for plant (CS-P), animal (CS-A), and fungal (CS-F) sequences. Sites integral in the decision tree analysis (DT) are also reported. Last, SWDA (S) and CVA (C) significant sites are shown within each factor score dataset (pah, pss, ms, cc, ec and all).

Further inspection of the basic region revealed that Animals shared several moderately conserved sites with Fungi. These shared sites included 2, 9, 10, and 12 where the most frequent amino acid was R, E, R, and R, respectively. Plants had three strongly conserved sites in the basic region, including sites 10, 12, and 13. Of these highly conserved Plants positions, site 13 was also highly conserved in Fungi, while sites 10 and 12 were only moderately conserved in Animals and Fungi.

In the first helix, many sites were highly conserved in all three kingdoms. These included sites 16, 23 and 27. Site 27 was the one of the most conserved in all three analyses with L being found almost exclusively at this position. Sites 16 and 23 were composed of hydrophobic amino acids I, L, V, and M. Site 20 was moderately conserved in all three kingdoms with the nearly the same set of amino acids (F, I, L, and M). Fungi shared conserved site 17 with Animals only, and site 28 only with Plants. Site 17 was only moderately conserved in Animals and Fungi with R being the most frequently occurring amino acid. Site 28, at the end of the first helix, P occurred at a frequency of 92%, 88%, and 73% for Fungi, Plants, and Animals, respectively.

We identified six conserved sites in the second helix, shared between Animals, Plants, and Fungi, including positions 53, 54, 57, 60, 61, and 64. The most frequently occurring amino acids in these sites were the same for all three Kingdoms (Figure 1). One of the most dramatic differences in conservation occurred at the beginning of the second helix (site 50), where animal and fungal sequences contained a K and plant sequences were much more variable. Finally, site 51 was the only conserved site shared exclusively between Plants and Animals, however, the most frequently occurring amino acids differed greatly, V, A, I, and L as compared to A, S, and V, respectively.

### Decision trees analysis

**Figure 2:**
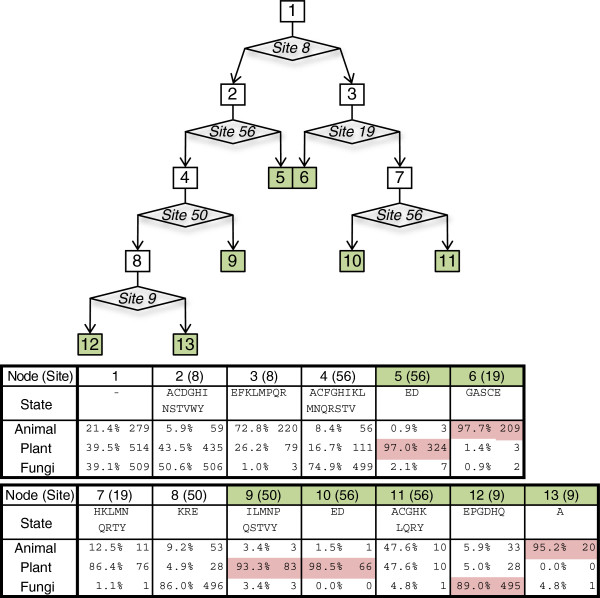
**Decision tree describing the classification of Plant, Animal, and Fungal bHLH sequences by amino acid sites found in the bHLH domain.** Each box of the figure represents a step in the decision tree which consist of a number of bHLH sequences from each Kingdom, and the amino acids at a given bHLH position (state). The sample size and proportion of group representatives is provided in the accompanying table. Diamonds contain the bHLH amino acid site which bifurcate the data into subsets of the previous state. Analysis is similar to a dichotomous taxonomic key.

Building a bifurcating decision tree based on observable characteristics has been the traditional method for classifying subjects [27]. Using the model bHLH sequences, we built a decision tree based on the amino acids at given sites of the domain (Figure 2). Starting from the entire 1302 sequence dataset, bifurcating steps were added until there were less than 10 sequences in a subset, the tree hit a depth of 4 steps, or the subset was almost completely comprised of plant, animal, or fungal sequences.

The resulting decision tree provided a straight forward method for classifying sequences, requiring only eight steps. At step 1, site 8 effectively discriminated animal sequences from plant and fungi, with the exception of animal groups D and E. Site 8 has previously been noted as a discerning site between animal groups [23]. Steps 2 and 4 split sequences based on sites 56 and 50, respectively. This separated Plants from Fungi and animal group D and E sequences. Finally, site 9 at step 8 split fungal sequences from animal groups D and E.

The decision tree accurately classified sequences as either Plants, Animals, or Fungi. It had accuracies of 95.6% for Plants, 95.8% for Animals, and 94.3% for Fungi with an overall accuracy of 92.8% (Table 2). Thus using only sites 8 and 9 (basic); 19 (Helix 1); 50 and 56 (Helix 2) the decision tree was able to discern Kingdom of origin for bHLH sequences with an accuracy over 92%.

**Table 2:** Validation of bHLH classified methods

**Kingdom**	**BestHit**	**Decision**			**SWDA**						**CVA**			
	**BLAST**	**Tree**	**pah**	**pss**	**ms**	**cc**	**ec**	**all**	**pah**	**pss**	**ms**	**cc**	**ec**	**all**
1302														
Plant	95.5	95.6	96.5	90.7	96.5	92.7	96.8	97.3	97.9	91.5	97.0	92.1	97.7	100
Animal	98.6	95.8	94.2	94.0	91.1	93.4	94.8	97.2	95.7	94.3	92.0	94.3	96.0	99.7
Fungal	98.0	94.3	92.9	91.6	90.4	90.6	94.6	95.8	94.0	90.7	90.8	90.7	95.0	99.6
Total	97.2	92.8	91.8	88.1	89.0	88.4	93.1	95.2	93.8	88.2	89.9	88.5	94.4	99.6
Unclassified	37	5	62	60	65	74	65	61	80	80	80	80	80	80
6987														
Plant	98.5	94.9	96.6	86.6	95.0	93.4	96.0	97.9	97.5	89.1	96.3	92.8	98.6	99.3
Animal	97.6	81.7	81.4	79.5	79.1	81.9	81.2	89.3	84.0	81.1	81.0	82.7	82.4	95.8
Fungal	97.3	82.7	82.0	84.8	81.6	84.1	82.4	89.2	84.6	85.4	82.3	85.3	82.6	96.1
Total	97.8	76.6	80.0	75.4	77.9	76.7	79.8	88.2	83.1	77.8	79.8	80.4	81.7	95.6
Unclassified	152	37	404	424	447	461	422	395	481	481	481	481	481	481

Note.-The accuracies are reported for several classification models; including, best hit BLAST, the decision tree analysis, SWDAS, and CVAS. The first measurements are based on the 1302 plant, animal, and fungal sequences used in building the models. The second set assesses the models with the 6987 sequence set which were not used in building the models. The number of sequences that were unable to be classified (Unclassified) for each model are also provided.

### StepWise discriminant analysis

To measure the discerning ability of individual amino acid sites at determining plant, animal, or fungal origin, we performed a StepWise Discriminant Analysis (SWDA) [22]. In order to perform this analysis, alphabetic amino acid data was transformed into numerical values by utilizing five numerical indices (factor scores). The five factor scores (**pah**, **pss**, **ms**, **cc**, **ec**) were based on measured physiochemical amino acid properties [23]. The transformation resulted in five numeric values for each amino acid at every position in each bHLH sequence. An additional factor score set (**all**) was created from the combination of all five factor scores. The result was a total of six factor score transformed datasets each containing 1302 numeric bHLH sequences.

To determine the most discriminating sites, numerical sequences were analyzed using the SWDA function in SAS denoted as SWDA{***factor score***}. The discriminating power of each site was evaluated by its partial correlation (*r*^2^) and the accumulated Average Squared Canonical Correlation (ASCC). Factor Scores **pah** and **ec** performed the best in SWDA, explaining 70% of the among-group variance in only 20 steps each. In contrast, Factor Scores **pss**, **ms**, and **cc** could only obtain average squared canonical correlations (ASCC) of 57%, 61% and 64% when using 36 amino acid sites, respectively. In the first three steps, **pah** and **ec** obtained 44% and 42% ASCC, respectively, and shared sites 2 and 13 within the top three discriminating amino acids.

When utilizing **all**, an ASCC of 80% was obtained using only 14 different amino acid sites within the 20 steps. The three most discerning sites, accounting for 49% of the among-group variability, were 5, 56 and 50. The most discerning site in this analysis, site 5, was transformed using the codon composition (**cc**) factor score. Sites 56 and 50 had been transformed by the **ec**, and **pah** factor scores, respectively.

StepWise models were built from SWDA at cutoffs described in the Material and Methods. The 1302 model sequence dataset was then classified by the StepWise classification models and the results recorded in confusion matrices (not shown). The models provided overall accuracies ranging from 88.1% - 95.2% for individual factor scores (Table 2). SWDA{**all**} was the most accurate (95.2%) of the stepwise analyses and utilized only 20 amino acid sites.

Amino acid sites with partial correlation (*r*^2^) > 20% were found to be highly diagnostic of plant, animal, or fungal bHLH origin (Table 1). SWDA using the five factor scores (**pah**, **pss**, **ms**, **cc**, **ec**), identified between 1–3 discerning sites for each analysis. With the most accurate Stepwise model, SWDA{**all**}, 4 sites (2, 5, 50, 56) were found to highly discerning.

### Canonical variate analysis

**Figure 3:**
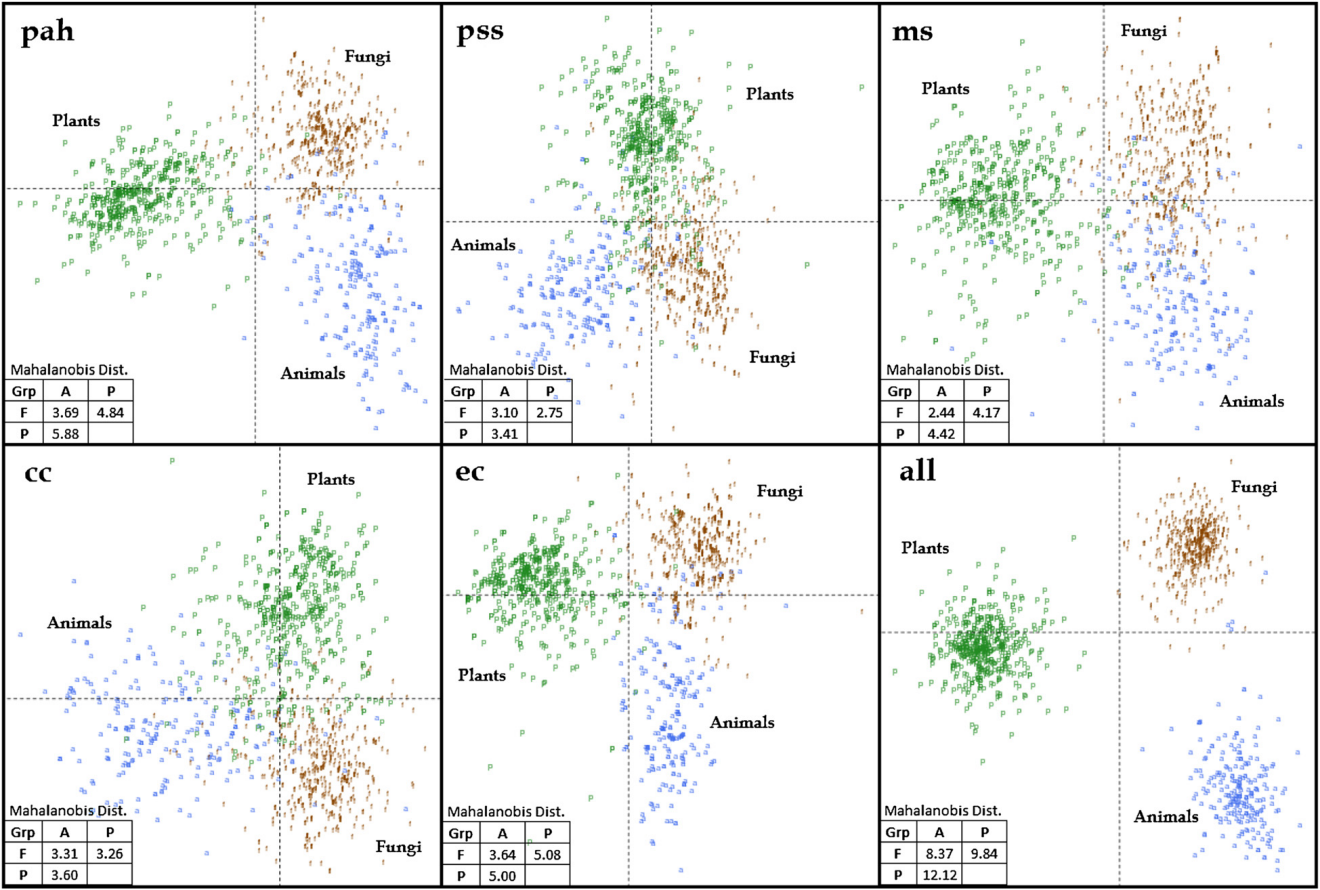
**Projection of PAF sequences onto canonical vectors for the factor score transformed datasets.** Each plot contains the two canonical variates from the CVA and the *sqrt* of the Mahalanobis pairwise distance between the centroids of Plants, Animals, and Fungi.

To leverage more discerning power, we utilized all bHLH amino acid sites simultaneously in a Canonical Variate Analysis (CVA) on the six numeric datasets, denoted CVA{***factor score***} [28,29]. Figure 3 shows the discerning power of each factor score set by plotting the two canonical variates (eigenvectors), along with the *sqrt* of the Mahalanobis pairwise distance between the centroids of plant, animal, and fungal sequences. The visual distinction and the Mahalanobis distance between plant, animal, and fungal groups was greatest in the CVA{**all**}. Although the Mahalanobis distance between Plants, Animals, and Fungi were smaller in **pah**, **pss**, **ms**, **cc**, and **ec**, the sequences clearly clustered among their Kingdom of origin in each separate analysis.

Both of the canonical variates (CV) in the factor score analyses were important in discerning between fungal, plant, and animal sequences (Table 3). The first CV in **pah**, **pss**, **ms**, **cc**, **ec**, and **all** explained 67%, 62%, 72%, 57%, 69%, and 62% of the total variation within their respective analyses. By default, the second CV accounted for the remaining unexplained variance. Fungi and Animals were separated from Plants by the first CV in **pah**, **ms**, **ec** and **all**; with the second CV discerning between Fungi and Animals. In the **pss** analysis, the first CV separated out Plants and Fungi from Animals; while in **cc**, Plants and Animals were first separated from Fungi.

**Table 3:** Discerning ability of canonical variates for each Canonical Variate Analysis

**Factor Score**	**CV**	**Var**	**Grps**	**Amino Acid Sites**	**all Amino Acid Sites**
pah	1	66.5%	P	50,54	9, 13, 20, 23, 50, 53, 57
	2	33.5%	A, F	13, 23	10, 16, 27
pss	1	61.9%	A	12, 13	6, 13, 20, 23, 54
	2	38.1%	P, F	23, 53	10, 23
ms	1	71.6%	P	20, 50	13, 20, 23, 54
	2	28.4%	A, F	12, 23	12, 13, 16, 23
cc	1	56.6%	F	5, 10	13
	2	43.4%	P, A	2, 50	10, 12
ec	1	69.1%	P	20, 27, 51, 54	9, 13, 16, 20, 23, 27, 51, 54, 57, 61
	2	30.9%	A, F	16, 20, 54	12, 20, 27, 51, 54
all	1	62.4%	P	*	-
	2	37.6%	A, F	*	-

NOTE.–The variance between plant, animal, and fungal sequences explained by each canoncial variate (CV) is reported. The type of sequences (Grps) that are separated by each CV is also shown. Within each CV, the top 2 amino acid sites (as ranked by weight) and those sites with weights >1 are provided. CV1 and CV2 in the all analysis had 27 and 16 discerning sites, respectively (all Amino Acid Sites).

Classification models were then built from the CVA and used to classify the 1302 model sequence dataset. Accuracies of the CVA classification models ranged from 90.7% - 99.6% for Fungi, 91.5% - 100% for Plants, and 92.0% - 99.7% for Animals (Table 2). CVA{**all**} was the most accurate model with an total accuracy over 99%. Thus, through CVA, we effectively distinguished bHLH domains from Fungi, Animals, and Plants.

In addition to the first CV of **pah**, **ms**, **ec**, and **all** separating Plants from Animals and Fungi, the Mahalanobis distances between animal and fungi bHLH domains were smaller in each CVA compared to the distances to plant domains. Taken together, these findings suggest, as expected, that Animals and Fungi are more closely related than they are to Plants [30].

Many of the top ranked sites of each CV, based on the magnitude of CV coefficients, were identified as major discerning sites in SWDA (Table 1). Sites 2, 5, 12, 13, 50 and 51 were identified by SWDA and CVA within the same factor score transformed datasets. For example, sites 2 and 5 were highly discerning when using the **cc** transformation. Likewise for **pss**, the most discerning sites were 12 and 13. Site 51 was discerning for the **ec** dataset in both analyses. Sites 13 and 50 in the **pah** data. While several sites were discerning for the **ms** dataset, none were shared by CVA and SWDA. Each of the most discerning sites had known molecular characteristics shared by the biological roles of the factor scores. Sites 2 and 5, discerning sites in **cc**, contact DNA and are crucial for correct E-box binding. Sites 12 and 13 are located at the transition from the secondary structures basic region to Helix 1. Last, sites 13 and 50 play crucial roles at the start of their helices, detected as discerning sites in the **pah** analyses. Thus, in addition to accurately discerning Kingdom origin, we linked highly discriminating sites to those with known molecular functions.

### Classification model testing

To evaluate the effectiveness of the different classification models in discerning between Kingdom origin, we determined and assessed the accuracy for a set of 6987 test bHLH domains (Table 2). This dataset was comprised of plant, animal, and fungal sequences not used to build models. In addition, we performed a BestHit BLAST analysis for comparative purposes. The overall accuracy for each test was above 86% for Plants, slightly lower at just over 79% for Animals and 82% for Fungi. BestHit BLAST and CVA{**all**} had accuracies greater than 95%. The CVA{**all**} model requires an amino acid in every position (no gaps) of the basic, Helix 1 and Helix 2 and could not classify 6.8% of the 6987 sequences. SWDA{**all**}, which used only 20 positions, was able classify a further 86 sequences that CVA{**all**} could not. While the CVA had a higher accuracy compared to SWDA (88.2%), SWDA may be preferable when CVA{**all**} cannot classify a given sequence. Both of these models are preferable to BestHit BLAST as they utilize site-specific conservation, provide a confidence in assigning each sequence to a Kingdom, and may be leveraged by additional statistical analyses. In contrast, BLAST only provides a measure of sequence similarity.

### BHLH origins from environmental samples

Classification of bHLH sequences of unknown origin is one possible application of these classification models. To test such an application, we obtained a publically available marine environmental sample with >6.125 million amino acid sequences [31]. We then ran InterPro Scan [32] to identify the presence of the bHLH domain in these sequences. This resulted in only 10 sequences being annotated with the IPR001092 signature. These 10 sequences were then extracted and classified using the SWDA{**all**} and CVA{**all**}. Additionally, we investigated Kingdom origin through the best BLAST hit for each sequence (Table 4). For 6 of the 10 sequences, CVA{**all**} and BLAST agreed with Kingdom origin. Three of the remaining four sequences could not be compared directly to the CVA{**all**} results as they had very low identity and coverage in the BLAST analysis. The hit with the best coverage and identity (ECU95545.1), was identified as an *Ostreococcus lucimarinus* (algae) sequence by BLAST. CVA{**all**} classified this same sequence as an animal, however, the Mahalanobis distance between the animal centroid and the algae sequence was greater than those between animal and plant centroids (Table 4, Figure 3). This distance is not unexpected as many algae have been shown to be highly divergent from other plant bHLH sequences [11], and only a handful of algae organisms were used to build the classification models.

**Table 4:** Classification of unknown bHLH sequences

**SeqId**	**BLAST**			**Mahal. Dist**			**Kingdom (Prob)**
	**Species Name (Common)**	**Cov.**	**iid**	**Animal**	**Fungi**	**Plant**	
ECU95545.1	*Ostreococcus lucimarinus* (green algae)	93%	90%	7.60	13.6	11.0	Animal
ECR14344.1	*Sus scrofa* (pig)	87%	32%	4.90	9.18	8.22	Animal
ECB02340.1	*Micromonas sp. RCC299* (green algae)	85%	68%	7.58	8.58	4.93	Plant
ECZ39823.1	*Ostreococcus tauri* (green algae)	79%	53%	9.14	6.53	3.51	Plant
EBY00838.1	*Monosiga brevicolli* (choanoflagellates)	49%	66%	3.34	8.02	14.3	Animal
EBG07815.1	*Methanocaldo-coccus vulcanius*	38%	29%	1.62	9.99	13.2	Animal
EBZ65459.1	*Branchiostoma belcheri* (Japanese lancelet)	36%	38%	2.31	10.2	12.1	Animal
ECI19714.1	*Daphnia pulex* (common water flea)	34%	44%	7.36	2.60	12.0	Fungal
ECP37863.1	*Aedes aegypti* (yellow fever mosquito)	30%	37%	4.63	4.81	8.19	Animal
EBW92904.1	*Cryptococcus neoformans* (basidiomycetes)	15%	38%	10.9	7.74	2.11	Plant

NOTE.–Ten unknown bHLH sequences from a marine environmental sample as identified by InterPro. The attributes of the best BLAST hit are reported (species, coverage, identity). The results of the CVA{all} are also reported along with the distance of the bHLH sequence to Plant, Animal, and Fungal Kingdoms. The predicted Kingdom of origin is also reported.

**Figure 4:**
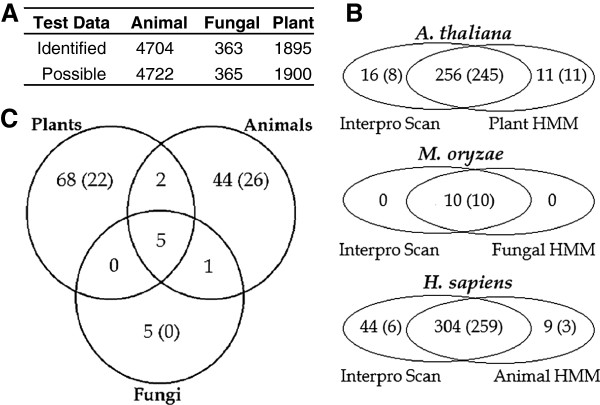
**Identification and classification of bHLH sequences from the test dataset and the marine environmental sample.A** The number of bHLH sequences found using Kingdom specific HMMs on the 6987 test dataset. **B** Identification and classification of bHLH sequences by Kingdom specific HMMs and CVA{**all**} as compared to Interpro Scan. Values in parenthesis represent the number of bHLH sequences classified into the same Kingdom as the respective organism. **C** Overlap of bHLH sequences found with the Kingdom specific HMMs on the environmental dataset. Values in parenthesis denote the number of bHLH sequences classified with CVA{**all**} as Plants, Animals or Fungi with respect to the Kingdom specific HMM.

To further explore the environmental sample, we built several HMMs using the alignment of the 1302 sequences in the expert data set. Each model identified >99.4% of bHLH sequences in the 6987 test data set (Figure 4A). To test the ability of the HMMs to accurately identify bHLH containing sequences, the complete proteomes of *Magnaporthe oryzae, Arabidopsis thaliana,* and *Homo sapiens* were scanned and compared to bHLH annotations from Interpro (Figure 4B). Using the appropriate Kingdom specific HMM, 10, 267, and 313 bHLH sequences were identified in *M. oryzae, A. thaliana*, and *H. sapiens* respectively. Within *M. oryzae*, the fungal HMM identified 10 bHLH sequences, the same set available with bHLH annotations from Interpro. The *A. thaliana* scan with the plant HMM identified 267 sequences, including 256 sequences out of 272 annotated by Interpro. Of the 267 bHLH sequences identified by the plant HMM, 256 were classified as Plant by CVA{**all**}. The *H. sapiens* analysis had the largest gap with Interpro, with Interpro identifying 44 sequences the animal HMM did not. However, of those 44, only 6 were classified as Animal by CVA{**all**}, 9 had fewer than 100 amino acids, and 1 had been deleted as a valid entry in Uniprot. Of the three model organisms scanned, *H. sapiens* had the fewest sequences classified correctly with only 84%.

Scanning of the Sargasso Sea environmental sample resulted in the identification of 125 sequences which were uniquely assigned to being of Plant (68), Animal (44), or Fungal (5) origin by the Kingdom specific HMMs (Figure 4C). Eight sequences were found by more than one Kingdom model, all of which were also identified by Interpro (Table 4). Although more than a third of the 117 sequences could not be assigned by our classification models, where an assignment could be made, the models largely supported Kingdom origin assigned by the HMM. For example, 26 of 44 sequences identified by the animal HMM were assigned as animal by CVA{**all**}. Blast analysis of the 117 sequences returned only 46 that had a significant match (>70% coverage and >70% identity) to a bHLH protein. These findings suggest that our HMMs are useful, in combination with our classification models, for determining the origin of bHLH domains in complex samples.

## Discussion

Using a decision tree, SWDA and CVA models, we found that only a few amino acid sites were necessary to classify bHLH domains by Kingdom. In the decision tree, only sites 8, 9, 19, 50 and 56 were needed to accurately classify sequences as Plant, Animal or Fungal. However, decision trees have many solutions and therefore do not always identify discerning sites. Thus, we used Discriminant analysis. SWDA and CVA were performed on the numerically (factor score) transformed bHLH data, which revealed 5 highly discerning sites in common, 2, 5, 12, 13, and 50 (Table 1). It is noteworthy that four of the five sites are in the basic region and all (including site 50) are known to contact either the phosphate backbone or the DNA based on animal bHLH crystalline structures [7,33,34]. From Figure 1, we also observed that the group entropies at these sites were distinct. For example, site 50 was highly conserved in Animals and Fungi, but more variable in Plants. These differences in conservation were characteristic of all highly discerning sites shared by CVA and SWDA.

As shown in Table 1, CVA typically identified discerning sites more readily than SWDA. For example, CVA, but not SWDA, identified a set of 6 amino acid sites spanning Helix 1 and Helix 2 that were highly discerning. Sites 16, 20, 23, 27, 53 and 54 were each determined to be discriminating by CVA{**all**}, and by at least one of the other CVA models. Each of these sites is important to the molecular architecture of the bHLH as buried sites within the helices. They are crucial for dimerization and partner specificity [6,35-37]. We also observed that each site was moderately to highly conserved in Plants, Animals, and Fungi. However differences in the degree of conservation between the Kingdoms were not clearly evident at each of these sites. Our findings suggest that the CVA model is more sensitive than SWDA to variations of conservation. One possible explanation for the lack of sensitivity of the SWDA model is that SWDA considers each site separately, whereas CVA considers all sites simultaneously.

We established that the decision tree, SWDA and CVA models were highly accurate using the model data set (Table 2). On the test dataset, the decision tree’s accuracy fell by more than 12% for Animals and Fungi, but remained high for Plants. SWDA{**all**} accuracy also declined by 8% for Animals and Fungi, while improving for Plants. Last, CVA{**all**} exhibited this same behavior, however the drop in accuracy for Animals and Fungi was only about 4%. As expected, accuracies declined using the test dataset, but overall remained high, particularly for Plants.

The discrepancy in accuracies between the tests may be due to underrepresentation of animal and fungal organisms in the model dataset. Fungal sequences in the model dataset only included bHLH proteins from completed genome projects, encompassing the Ascomycota and Basidiomycota phylums. Other phylums, such as Zygomycota and Chytridiomycota, were not represented in the fungal model dataset. Model animal sequences, obtained from published work, are known to be highly representative of higher order Metazoans. However, this set of animal model bHLH domains lacks sequences from ancestral (early Metazoan) organisms. The higher accuracies for Plants using the test dataset may reflect a lack of variation in the available sequences, in particular a lack of sequences from algae, as evidenced by the inability to classify an algae sequence in the environmental sample (Table 4). Upon further examination, this sequence was quite distant from any group. Indeed, the Mahalanobis distance between this sequence and the centroid for Plants, Fungi or Animals was greater than the distance between centroids. It is likely this sequence may have been classified as Plant if more algae sequences were available to incorporate into the model dataset.

From our testing of classification models, it was evident that CVA{**all**} was the most accurate of all the analyses (Table 2, Figure 3). However, CVA{**all**} does not tolerate missing data (gaps), and could not classify nearly 7% of the test sequences. Since SWDA{**all**} requires less discerning sites, it was more tolerant of missing data, classifying 86 sequences that CVA could not. Thus, even with lower accuracies, SWDA is the preferable classification model in instances of missing data.

Accuracy measurements established the effectiveness of the classification models. However, the CVA and SWDA models are not simply designed to classify sequences, but they elucidate the underlying architecture of the bHLH domain (discerning sites) (Tables 1, 3). Other classification methods, such as phylogeny or BLAST based methods, may accurately classify sequences to correct Kingdom origin as we report here (Table 2). While CVA{**all**} and BestHit BLAST are highly accurate, BLAST ignores the fundamental attributes of the bHLH domain. Furthermore, CVA and SWDA models have been used to classify highly divergent sequences that BLAST had failed to identify (Table 4) [20].

The models we have developed are valuable for identification of bHLH domains of unknown origins within large datasets, such as an environmental sample (Figure 4). Using InterPro Scan, only 10 bHLH sequences were identified. However, 117 additional bHLH sequences were identified by our Kingdom specific HMMs, 48 of which were classified into Kingdom of origin with two lines of evidence (Kingdom specific HMM and CVA{**all**}). On the other hand, BLAST analysis only retrieved 46 of these sequences, again highlighting another advantage of using models rather than sequence similarity for identifying bHLH sequences. One explanation for finding 117 additional sequences using the Kingdom specific HMMs, in contrast to Interpro Scan, is that variation between the Kingdoms has been partitioned out. Thus, the Interpro model may be considered too generalized, resulting in lower probability scores for diverse bHLH sequences.

CVA{**all**} with the 125 Kingdom specific HMM selection and the 10 Interpro Scan selections, classified twice (or more) as many Animal and Plant sequences as Fungal (Figure 4). This may be due to the sample containing fewer fungal organisms or may be a reflection of the sampling technique used to collect the sample (i.e. passing the sample through a 10 μm filter). Marine fungi are uncommon in open sea water, being typically associated with substrata such as submerged timbers and organic rich sediment [38].

The environmental sample contained 6.125 * 10^6^ protein sequences, yet our scans only found 10–125 contain the bHLH domain. This is likely due to a combination of many factors, including; 1) 1.2 * 10^6^ sequences contained less than 100 amino acids, 2) sequencing and assembly errors, 3) incorrect translations of DNA, and 4) 10 μm filter removing the majority of organisms that are represented in the model building set.

In addition to the utility of the Kingdom specific models for scanning environmental samples, they also identify additional bHLH sequences within genomes. We identified an additional 9 bHLH sequences within *Homo sapiens* and 11 in *Arabidopsis thaliana* (Figure 4). CVA{**all**} provided further evidence of correct Kingdom origin for all the sequences from *Arabidopsis thaliana* and 3 from *Homo sapiens*. Furthermore, our findings strongly suggest that other organisms may contain unidentified bHLH proteins. Thus, it is likely that our models will identify previously unreported bHLH sequences in characterized genomes.

It is important to note that our classification models will only classify sequences as Plant, Animals or Fungi. Posterior probabilities provided by the models may potentially be misleading as these are the probability a sequence is either Plant, Animal or Fungal given those are the only options. Thus, bHLH sequences from other Kingdoms, such as Chromista (Stramenopiles) and Protozoa (Amoebozoa) will be misclassified as either being Plant, Animal, or Fungal. Therefore, it is prudent to examine the Mahalanobis distance to determine classification strength. These Kingdoms were not included in this work as they do not have enough bHLH sequences to adequately train a discriminant model. However, as additional bHLH sequences become available with the recent onslaught of genome projects, inclusion of additional Kingdoms in the model would likely increase the effectiveness of environmental sample classification.

From Figure 1, we can easily note the differences in conservation of the bHLH domain between the Kingdoms, especially in the basic region. Based on the consensus sequence alone, it is evident that Plants have a more variable basic region, while Animals and Fungi are more conserved [18]. Also, Fungi have greater conservation at sites 9–13 than either Plants or Animals. Differences in conservation are evident in Helix 1 and Helix 2 as well, with the most dramatic occurring at site 50 where Plants are much less conserved. These differences are important to note as they suggest differences Kingdom level selection pressure on this domain. Previous work done by Atchley et. al. [23] found different patterns of selection on Animal binding groups. Additionally, Sailsbery et. al. [20] found varying selection patterns on Fungal groups that correlated directly with taxonomy. Thus, we find the most likely explanation for our highly discerning and biologically meaningful amino acid sites are that there exist majority Kingdom specific selective pressure that is evidenced in their bHLH architecture.

## Conclusions

Discriminant analyses can directly associate biologically meaningful amino acid sites with apriori defined groups (Kingdoms, Phylums, binding groups, etc.), when those analyses are built from factor score transformed peptides. In this manner they can be used to elucidate the underlying architecture of proteins of interest. Such models can also be used to classify large sets of highly divergent data quickly and accurately. We leveraged both of these strengths in our work. We were able to identify slightly divergent sites, each tied directly to the architecture of the bHLH domain, differentiated by Kingdom, across vast distances of evolutionary time. Furthermore, we demonstrated that our classification models, in conjunction with Kingdom specific HMMs, quickly and accurately classified sequences from large datasets, such as unknown bHLH sequences from an environmental sample. Furthermore, our models identified additional unreported bHLH domains within characterized genomes. All models and data have been incorporated into an open source online tool at http://www.fungalgenomics.ncsu.edu.

## Methods

A set of 1302 expertly aligned and enumerated bHLH was compiled from previous work [11,20,23,24]. This set was used to build classification models and included 514 plant, 279 animal, and 509 fungal sequences (Additional file 1: Table S1). A bHLH sequence dataset for testing classification models was generated as follows. First, protein sequences containing the IPR001092 signature were identified using Interpro 31.0 [39]. Next, using an iterative alignment [20], each bHLH domain was enumerated comparable to the expert build set. Last, bHLH sequences were extracted and placed into the test dataset. The resulting test dataset was comprised of 6987 bHLH plant (1900), animal (4722), and fungal (365) sequences (Additional file 1: Table S1).

The Boltzmann-Shannon entropy value [40] was calculated for each site in the build sequence alignment for: 1) plant sequences, 2) animal sequences, and 3) fungal sequences. To determine the normalized group entropy value: 1) amino acids were grouped based on their physicochemical properties (acidic, basic, aromatic, aliphatic, aminic, hydroxylated, Cysteine, Proline) resulting in eight sets (DE, HKR, FWY, AGILMV, NQ, ST, C, P, respectively) [8,41]; 2) The Boltzmann-Shannon entropy value, based on the eight amino acid groups, was calculated at each site [40]; 3) These group entropy values were normalized to range from 0 to 1, with respect to possible minimum and maximum values, respectively.

Consensus sequences were determined by using the *5010* rule [13]. For a given site, an amino acid was included in the consensus sequence if it had a concentration >50% across the entire sequence alignment. For each incorporated *50* site, every amino acid with a concentration >10% was also included. Using these rules, the animal consensus motif was adapted from previous work [8]. Fungal and plant consensus motifs were taken from previously published work [13,20].

### Classification models

Decision Trees [23,27] were built using SAS software, Enterprise Miner 5.2. A Chi-square test with a significance level of 20% was used as the splitting criteria. The bifurcating tree was limited to a depth of 4 nodes, requiring a minimum of 10 observations for a split and at least 4 observations per leaf. Best hit BLAST (BLASTP) [42] were the non self identified best hits for each bHLH sequence against the NR database with >80% identity and an e-value < .01.

Alphabetic amino acid data from the 1302 bHLH sequence alignment was transformed into a 1 × 5 vector of numeric values using the HDMD package [43]. Transformation to numerical values used independent factor scores which are quantitative values for amino acids based on 495 amino acid properties [23]. The five factor scores (**pah**, **pss**, **ms**, **cc**, **ec**) are associated with the biological properties: polarity, accessibility, and hydrophobicity; propensity for secondary structure; molecular size or volume; codon composition; and electrostatic charge; respectively. We created an additional dataset containing the combination of all five factor scores (**all**). This resulted in the total of six factor score transformed datasets: **pah**, **pss**, **ms**, **cc**, **ec**, and **all** from the 1302 bHLH sequences.

Canonical Variate Analysis (CVA) and StepWise Discriminant Analysis (SWDA) [22] were used to build classification models on all six factor score datasets. These discriminant analyses defined the latent structure of covariation among the Kingdoms and identified the sites that best differentiated between plant, animal, and fungal sequences.

The step-up SWDA procedure ranked amino acid sites based on their ability to discriminate Plants, Animals, and Fungi, as measured by the Wilks’ lambda likelihood ratio [23]. In the step-up procedure, variables (amino acid sites) were added sequentially (steps) based on Wilk’s lambda, until an Average Squared Canonical Correlation (ASCC) reached a value of 70% for **pah**, **pss**, **ms**, **cc**, **ec** and 80% for **all** datasets (Additional file 1: Table S2). The ASCC describes the related distinctiveness of the groups at a given step in the model, meaning a 100% ASCC would imply complete discrimination between the defined groups. The partial correlation (*r*^2^) was also measured, which is a measure of each site’s ability to discriminate between groups while controlling for the effects of other variables already in the model. Those variables with *r*^2^ > 20% were considered the most discerning sites. SAS software, Version 9.2 was utilized in the SWDA.

CVA assesses the discriminatory ability of all variables simultaneously to generate a linear model to differentiate between defined groups. The CVA includes the calculation of eigenvectors (canonical variates) from the among-group covariance matrix. CVA for the six factor score datasets resulted in 2 canonical variates for each analysis (Additional file 1: Tables S3 & S4). The square root of the Mahalanobis pairwise distance was also calculated, providing a relative measure of the divergence between groups. CVA and plotting of canonical variates were conducted utilizing the statistical software package **R**[44], specifically the *lda* function as described in the HDMD package [43]. Amino acid sites were considered discerning if they had absolute magnitudes >1 in either canonical variate for the **pah**, **pss**, **ms**, **cc**, **ec**, and **all** CVAs.

### Hidden Markov models

Hidden Markov Models (HMM) were built using HMMER 2.3.2 [45,46]. The 1302 model dataset was first divided by Kingdom. HMMs were then separately constructed on the first and second helix for sequences from each Kingdom. There were three Kingdom specific HMMs (Plant Helix 1 – Plant Helix 2; Animal Helix 1 - Animal Helix 2; Fungal Helix 1 - Fungal Helix 2). Sequences were identified as bHLH if and only if a Helix 1 hit completely preceded Helix 2 hit and both helices had scores greater than 0.1.

### Testing methods

The performance of classification models was determined by comparing predicted Kingdom origin to the actual origin in the 7692 test sequence set [20]. Positive and negative classification results were recorded for each model in 3×3 confusion matrices (data not shown) where sensitivity and specificity were measured using the *One versus All* approach [47]. The overall ability of models to correctly identify results was then determined (accuracy).

### Environmental sample

Translated amino acid sequences from the Sargasso Sea marine sequencing project [31] were provided by JCVI (http://www.jcvi.org). This set included 6.125 * 10^6^ putative protein sequences. The sequences were scanned by InterPro Scan installed on compute clusters provided by the NC State Office of Information Technology High Performance Computing (http://www.hpc.ncsu.edu). BLAST [42] analyses were performed on selected environmental sequences using the non-redundant NCBI database, e-value threshold of 10, word size of 3, and the BLOSUM62 matrix.

## Abbreviations

DNA: Deoxyribose Nucleic Acid; bHLH: Basic Helix-Loop-Helix; PAS: Per-Arnt-Sim; CVA: Canonical Variate Analysis; SWDA: StepWise Discriminant Analysis; ASCC: Average Squared Canonical Correlation; CV: Canonical Variate; pah: Polarity, accessibility and hydrophobicity; pss: Propensity for secondary structure; ms: Molecular size; cc: Codon composition; ec: Electrostatic charge; COE: Collier/Olf-1/EBF; HMM: Hidden Markov Model; *r*^2^: Partial correlation; BLAST: Basic Local Alignment Search Tool.

## Competing interests

The authors declare that they have no competing interests.

## Authors’ contributions

JS carried out the analyses, experimental design, tool construction, and drafted the manuscript. RD provided insight of data results, directed research, was essential in editing and drafting the manuscript, and provided funding for the project. Both authors read and approved the final manuscript.

## Supplementary Material

Additional file 1 An excel file that contains Supplementary Tables S1-S4. Click here for file
